# Comparison of postoperative pain between patients who underwent primary and repeated cesarean section: a prospective cohort study

**DOI:** 10.1186/s12871-019-0865-9

**Published:** 2019-10-22

**Authors:** Guangyou Duan, Guiying Yang, Jing Peng, Zhenxin Duan, Jie Li, Xianglong Tang, Hong Li

**Affiliations:** Department of Anesthesiology, Xinqiao Hospital, Army Medical University, Chongqing, 400037 China

**Keywords:** Cesarean section, Postoperative pain, Analgesia, Primiparas, Multiparas

## Abstract

**Background:**

The differences in post-operative pain are unclear between the primiparas who underwent a primary cesarean section and multiparas who underwent their first repeat cesarean section. The study aimed to explore the possible differences in postoperative pain between primiparas and multiparas.

**Methods:**

A prospective cohort study was performed only including women who underwent cesarean deliveries under spinal anesthesia. Postoperative patient-controlled intravenous analgesia (PCIA) was administered to all subjects with 0.2 mg/kg hydromorphone and 4 mg/kg flurbiprofen; the pump was programmed as 2.0 mL/h background infusion with a loading dose of 1 mL and a lockout period of 15 min. Postoperative incision and visceral pain intensity were evaluated using the visual analogue scale, and inadequate analgesia was defined as a visual analogue scale score ≥ 40 during 48 h post-operation. Additionally, the patients’ pain statuses in postoperative week 1 and week 4 were also assessed during follow-up via telephone.

**Results:**

From January to May 2017, a total of 168 patients (67 primiparas and 101 multiparas) were included. The relative risk for multiparas to experience inadequate analgesia on incision pain was 0.42 (95% CI: 0.25 to 0.74) compared to primiparas. In patients aged < 30 years, inadequate analgesia on visceral pain was higher in multiparas than in primiparas (RR, 3.56 [1.05 to 12.04], *P* = 0.025). There was no significant difference in the combined incidence of inadequate analgesia in both types of pain between the multiparas and primiparas (33.7% vs. 40.2%, *P* = 0.381). No difference was found in PCIA use between the two groups (111.1 ± 36.0 mL vs. 110.9 ± 37.3 mL, *P* = 0.979). In addition, a significantly higher incidence of pain was noted 4 weeks post-surgery in primiparas than that in multiparas (62.2% vs. 37.7%, *P* = 0.011).

**Conclusion:**

Multiparas who underwent their first repeat cesarean section have a lower for inadequate analgesia on incision pain during the first 48 h after surgery than primiparas. Multiparas aged under 30 years may be more prone to experiencing postoperative inadequate analgesia on visceral pain.

**Trail Registration:**

ClinicalTrial.gov: NCT03009955, Date registered: December 30, 2016.

## Background

Cesarean section is the most common impatient surgical procedure globally. In 2016, the cesarean delivery rate in the United States was 31.9% [1]. In China, the annual cesarean delivery rate reached 41.1% in 2016 after relaxation of the one child policy [2]. However, despite the numerous measures that have been developed to manage postoperative pain, inadequate analgesia after cesarean section is common, with an incidence of nearly 50% [3–6]. Therefore, post-operative pain treatment remains a considerable clinical challenge in acute postoperative care during cesarean section. Inadequate postoperative pain management is associated with persistent pain, delayed functional recovery, and a longer hospital stay, which increase medical expenses, and is becoming a public health issue [7, 8]. Therefore, the treatment of pain after a cesarean section remains unresolved.

In China, a new clinical challenge for the treatment of pain after a cesarean section has emerged, following the implementation of China’s new national two-child policy [9, 10]. Many obstetric patients with known history of previous cesarean section are scheduled to undergo repeated cesarean section. Because repeated cesarean sections is common in very aged individuals and are known to have higher operative difficulties and longer surgical times due to severe adhesions [11, 12], we speculated that there would be a difference in pain control during the postoperative period between the patients who underwent repeated and primary cesarean sections; and that the multiparas may have a higher risk of receiving inadequate analgesia.

Intravenous or intrathecal analgesia with opioids is recommended and is a commonly used method for pain treatment after cesarean delivery. However, currently, most female patients receive a one-size-fits-all approach for analgesia after cesarean section, regardless of primiparas or multiparas. In the recent Practice Guidelines for Obstetric Analgesia and Anesthesia, there was no specific explanation for the possible difference in postoperative pain between the patients who underwent repeat and primary cesarean sections [13, 14]. There are limited studies focusing on this issue. In addition, exploring the inter-individual variability in the degree of pain, and accurately targeting treatment in women who may experience inadequate analgesia may improve clinical outcomes [15, 16]. Therefore, the current prospective cohort study included patients who were scheduled to undergo primary or repeated cesarean sections to investigate the potential difference in postoperative pain between them.

## Methods

### Patients

This study was conducted according to the STROBE recommendations [17, 18]. The study protocol was approved by the Institutional Ethics Committee of Xinqiao Hospital, Third Military Medical University, Chongqing, China. Prior to the enrollment of patients, written informed consent was obtained from all the patients, and the study was registered on Clinicaltrial.gov (ID: NCT03009955).

**Fig. 1:**
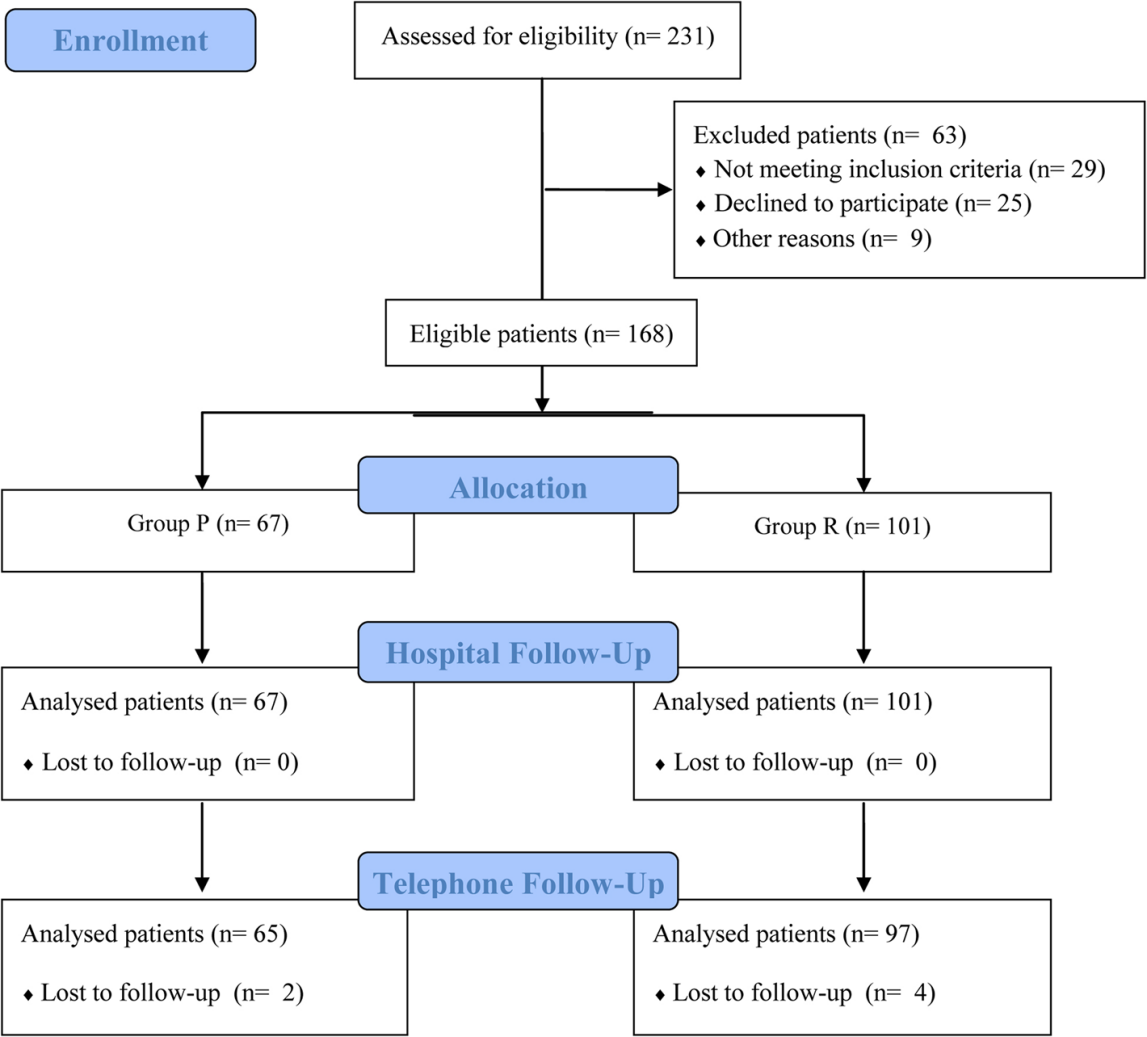
The flow diagram of the study

Patients were included according to established inclusion and exclusion criteria. From January to May 2017, 168 Chinese patients, aged 20 to 40 years scheduled to undergo elective cesarean section with a transverse incision were recruited for this study (Fig. 1). Patients who had a gestational age of 37 to 40 weeks and singleton pregnancy, voluntarily receiving intravenous patient-controlled intravenous analgesia (PCIA) treatment, and classified as having ASA physical status scale I-II were eligible for participation. The reasons for an elective cesarean section in a primipara included the patient’s own choice, preoperative complications including malpresentation (breech and transverse positions and compound presentation), placenta previa, uterine inertia, gestational diabetes, chronic or gestational hypertension, and preeclampsia. For the multiparas, the indication for a cesarean section was a previously scarred uterus. Only those who were undergoing their first repeat cesarean deliveries were included. Exclusion criteria included a history of chronic pain disorder, recent or chronic opioid use, substance abuse, heavy smoking (> 30 pack-years) [19] or alcohol dependence, absolute or relative contraindication to subarachnoid space block anesthesia, history of prior pelvic or abdominal surgery, or severe pregnancy complications, such as heart disease, brain disease, liver disease and kidney disease, that were life-threatening and required emergency treatment prior to the cesarean section.

### Anesthesia and Analgesia Management

Cardiac rhythm via electrocardiography, mean arterial pressure, and pulse oxygen saturation were monitored after the patients entered the operating room. Standardized anesthesia was administered by an experienced anesthetist, and the operations were conducted by a single surgical team using the same standardized technique. Spinal anesthesia, via a subarachnoid space block at the L3–4 interspace, was administered using 0.66% ropivacaine (20 mg).

After the fetal section and once daily after the surgery, oxytocin (20 IU in 500 mL of saline) was routinely administered while the patient was admitted to the obstetrics ward. PCIA was started immediately after surgery with a mixture of hydromorphone (0.2 mg/kg), flurbiprofen (4 mg/kg), and 0.9% normal saline at a dose volume of 200 mL, using a controlled infusion pump. The pump was programmed to a loading dose of 2 mL, background infusion rate of 2.0 mL/h, and PCIA dose of 1 mL, with a lockout period of 15 min. For the prevention of the postoperative nausea and vomiting, 3 mg of droperidol was administered at the outset of PCIA. Patients were monitored for 6 h in the postanesthesia care unit of the obstetrics ward after surgery. When pain was treated inadequately, the patients were administered additional pain treatment with tramadol 50 mg in a timely manner.

### Outcome Measurements

Standardized training for follow-up assessment was performed for all included investigators. A pain visual analogue scale (VAS score; 0–100, where 0 is defined as no pain and 100 as maximum pain) was used to evaluate postoperative pain at 4, 8, 12, 24, and 48 h. The primary outcome was the incidence of inadequate analgesia (defined as a pain VAS score ≥ 40) [20] during the postoperative 48 h. Pain caused by abdominal incision at rest and during mobilization (during coughing) was assessed using the VAS. Visceral pain was also assessed using the VAS. For visceral pain, the subjects were asked to report the pain induced by uterine contractions and were informed that the visceral pain could be enhanced when oxytocin was given. The duration of pain according to the patient’s self-reported time and PCIA consumption for 48 h after surgery were recorded.

Assessment with the hospital anxiety and depression scale (HADS) before the operation was performed in all patients. The HADS includes 14 assessments, including the symptoms of anxiety and depression (seven items scored 0 to 3 in each subscale, yielding a range of 0–21) with subscale scores of 8 indicating possible anxiety or depression [21, 22]. The intraoperative amount of blood loss, neonatal Apgar score, weight and height of the newborn, and surgery time were recorded. The Ramsay sedation score, respiratory rate, pulse oxygen saturation, systolic pressure, diastolic pressure, and heart rate were recorded before surgery and during the postoperative 48 h. Early walking time (determined by the time point when patients could ambulate) was also recorded. The sleep quality (rated as good or poor) on the day of and 1 day after surgery was evaluated. Postoperative adverse events including nausea, vomiting, and pruritus were also noted. Additionally, the patients’ duration of hospital stay was recorded.

The results of routine blood examinations before and 24 h after the surgery were retrospectively collected for all patients. The leukocyte and neutrophil counts were analyzed. At 1 week and 4 weeks after surgery, patients were interviewed by telephone and asked the following questions from a standardized questionnaire: Was there an existing pain? Was the location of pain at the incision, viscera, both, or none? Was sleep affected? Were they able to perform the activities of daily life with full autonomy, partial dependency, or absolute dependence?

### Sample size determination

The sample size was calculated according to the design of chi-square test for four-fold table data in a cohort study. Since previous studies reported the incidence of moderate to severe pain under postoperative analgesia for primipara as approximately 50% [4, 5], the current study hypothesized that the relative risk (RR) value for multiparas was 1.5 compared to that for primiparas. The anticipated incidence for multiparas was 75%. Therefore, based on a significance level of 0.05, power of 0.9, and an estimated ratio between the number of multiparas and primiparas of 1.5, according to the retrospective analysis based on the data from our hospital Electronic Medical Records System of the past 1 year, and considering about 3% loss of follow-up, the total required minimum sample size was determined to be 168 individuals using the sample size calculation software PASS, version 11.0 (NCSS, Kayesville, UT).

### Statistical Analysis

Statistical analysis was performed using SPSS for Windows version 19.0 (SPSS Inc., Chicago, IL). A two-tailed *P*-value less than 0.05 was considered statistically significant. The mean ± standard deviation (SD), median (interquartile range), and number (frequency) were used to summarize the variables. The patients who were scheduled to undergo a primary cesarean section were designated as group P in the final analysis, while the patients scheduled to undergo repeat cesarean section were designated as group R. The primary outcomes (postoperative inadequate analgesia on incision or visceral pain) were respectively described and analyzed. Logistic regression analysis using enter model was performed to evaluate the role of group P or group R in the prediction of postoperative inadequate analgesia. The presence of postoperative inadequate analgesia on incisional and visceral pain was considered as the outcome variable. BMI, age, gestational age, surgery time, preoperative complications (yes/no), depression (yes/no), and anxiety (yes/no) were also considered in the model. Odds ratios (OR) with 95% confidence intervals (CIs) were determined based on the logistic regression analysis.

An independent-sample t test was used to compare the differences in demographic and preoperative data between group P and R. Due to abnormal distribution, HAD scale, incision pain VAS at rest, and visceral pain VAS were compared using a Mann-Whitney U test. Propensity score matching (PSM) analysis was performed using STATA version 12 (Stata Corp, College Station, TX). Group P and group R were matched by propensity scores, and factors used to generate the propensity scores were those preoperative factors which had significant difference between the two groups. These factors included age, gestational age, and preoperative complications. Patients were matched in a 1:1 ratio without replacement. The caliper was defined as 0.2. The absolute standardized difference was calculated, and the absolute standardized difference less than 10% was considered to support the assumption of balance between the two groups. Then, other postoperative outcomes including the start time to feel pain, early walking time, hospital stays and PCIA administration were compared between groups P and R.

Differences in the incidence of postoperative inadequate analgesia, sleep quality, adverse events, and long-term pain status between the two groups were analyzed using Pearson’s chi-squared test. Furthermore, RR values and 95% CI for the probability of the occurrence of inadequate analgesia on incision pain and visceral pain during the postoperative 48-h follow-up were calculated, as well as the postoperative pain status at 1 and 4 weeks. Subgroup analysis according to age group (≤30 years or > 30 years) was performed. Two-way repeated analysis of variance (ANOVA) with post hoc LSD testing was used to compare the preoperative and postoperative systolic pressure, diastolic pressure, heart rate, respiratory rate, and leukocyte and neutrophil counts between the two groups.

## Results

### General results

Among the 67 primiparas who were scheduled to undergo cesarean section, 54 underwent the procedure due to preoperative complications (maternal or fetal factors) and 13 due to social factors. For the 101 multiparas, all underwent the procedure due to the history of a previous cesarean section. Fifty-four also had accompanying preoperative complications. As shown in Fig. 1, all patients completed the postoperative 48-h follow-up. However, six patients (two in group P and 4 in group R, *P* = 0.739) could not complete the study either because they could not be contacted or they withdrew from the study. The demographic and preoperative data of all patients are shown in Table 1. The results showed that the incidence of severe bleeding (≥ 500 mL) was 5.9% (6/101) in group R and was 7.5% (5/67) in group P and that there was no difference between the two groups (*P* = 0.696).

**Table 1:** Demographic, preoperative and intraoperative data

	Group P (*n* = 67)	Group R (*n* = 101)	Statistics
Age (year)	29.5 ± 3.9	31.3 ± 3.4	*t* = 3.112, *P* = 0.002
Age group (> 30)	20 (29.9%)	57 (56.4%)	χ^2^ = 11.467, *P* = 0.001
BMI (kg/m^2^)	26.7 ± 1.9	26.9 ± 1.9	*t* = 0.820, *P* = 0.415
Gestational age (week)	38.9 ± 0.9	38.4 ± 0.6	*t = 103*, *P* < 0.001
Preoperative complications	54 (80.6%)	54 (53.5%)	*t* = 12.912, *P* < 0.001
HADS-A(score)	2 (0, 5)	1 (0, 4)	*U* = 0.887, *P* = 0.375
HADS-D (score)	0 (0, 2)	0 (0, 2)	*U* = 0.129, *P* = 0.897
Surgery duration (min)	62.1 ± 15.3	71.1 ± 16.2	*t* = 3.782, *P* < 0.001
Weight of newborn (g)	3278 ± 481	3443 ± 1074	*t* = 1.185, *P* = 0.238
Height of newborn (cm)	49.7 ± 2.1	49.9 ± 1.7	*t* = 0.820, *P* = 0.505
Blood loss (mL)	286 ± 94	306 ± 92	*t* = 0.668, *P* = 0.889

Group P and R mean patients who received primary and repeated cesarean delivery, respectively; Data were presented as Means±SD, median (interquartile range) or as numbers (percentage); *BMI* = Body mass index; *HADS-A* = Hospital anxiety scale; *HADS-D* = Hospital depression scale

### Logistic regression analysis

Enter logistic regression models were applied to explore the possible predictors for postoperative inadequate analgesia on incisional pain and visceral pain. For the model of incisional pain, the statistical test for the overall model was significant (*P* = 0.001), and the predicted accuracy rate based on this model was 80.8%, while the overall model was not significant (*P* = 0.589) for visceral pain. As summarized in Table 2, patient group and preoperative complications were identified as significant factors for inadequate analgesia on incision pain. This showed that patients in group P or with accompanying preoperative complications would have higher odds of inadequate pain control.

**Table 2:** Logistic regression analysis of inadequate analgesia on incision pain and visceral

Outcome	Predictors	Wals	*P* value	OR	95% CI
Inadequate analgesia on incision pain	Age (year)	0.543	0.461	1.043	0.932 to 1.169
	BMI (kg/m^2^)	0.193	0.660	1.048	0.850 to 1.293
	Gestational age (week)	0.035	0.853	1.048	0.637 to 1.727
	Preoperative complications (yes/no)	4.721	0.030	0.365	0.147 to 0.906
	Surgery duration (min)	3.610	0.057	1.000	0.999 to 1.000
	Patient group(P/R)	10.790	0.001	0.191	0.071 to 0.513
	Anxiety (yes/no)	0.000	0.999	0.000	NA
	Depression (yes/no)	0.000	0.999	0.000	NA
Inadequate analgesia on visceral pain	Age	3.463	0.063	0.897	0.801 to 1.006
	BMI	0.002	0.968	1.005	0.804 to 1.256
	Gestational age (week)	0.175	0.675	0.885	0.498 to 1.570
	Preoperative complications (yes/no)	0.277	0.599	1.273	0.519 to 3.124
	Surgery duration (min)	0.586	0.444	1.000	0.999 to 1.000
	Patient group(P/R)	0.599	0.439	1.515	0.529 to 4.340
	Anxiety (yes/no)	0.133	0.715	1. 462	0.190 to 11.219
	Depression (yes/no)	0.423	0.515	0.403	0.026 to 6.228

*BMI* = Body mass index; *CI* = Confidence interval; *OR* = Odds rate

### Postoperative Data

**Fig. 2:**
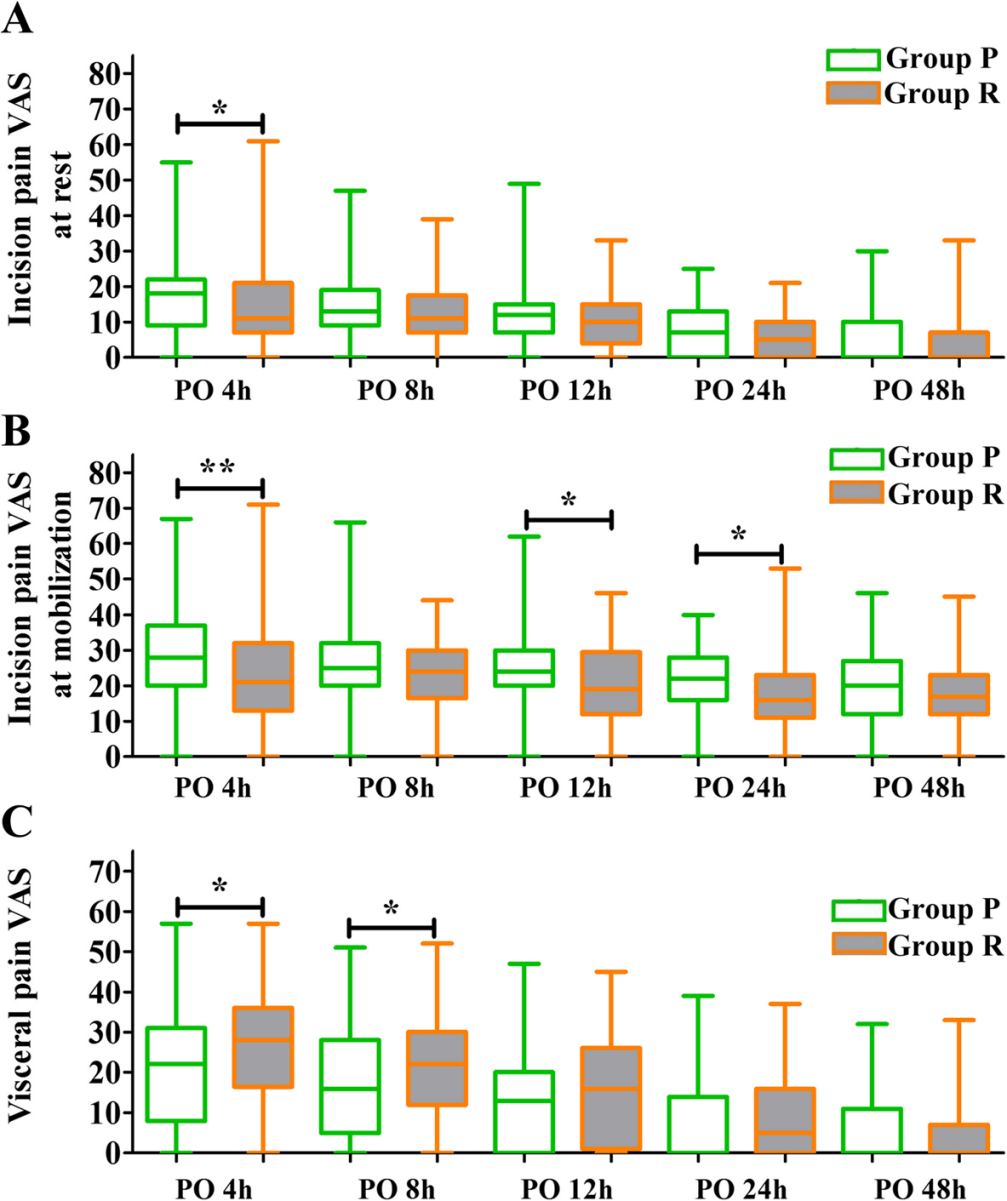
The distribution of postoperative incision pain VAS at rest (**a**) incision pain VAS at mobilization (**b**) and visceral pain VAS (**c**) at different time points. Means of groups P and R patients who received primary and repeated cesarean section, respectively; VAS = visual analogue scale; PO = postoperative; * *P* < 0.05; ** *P* < 0.01

**Fig. 3:**
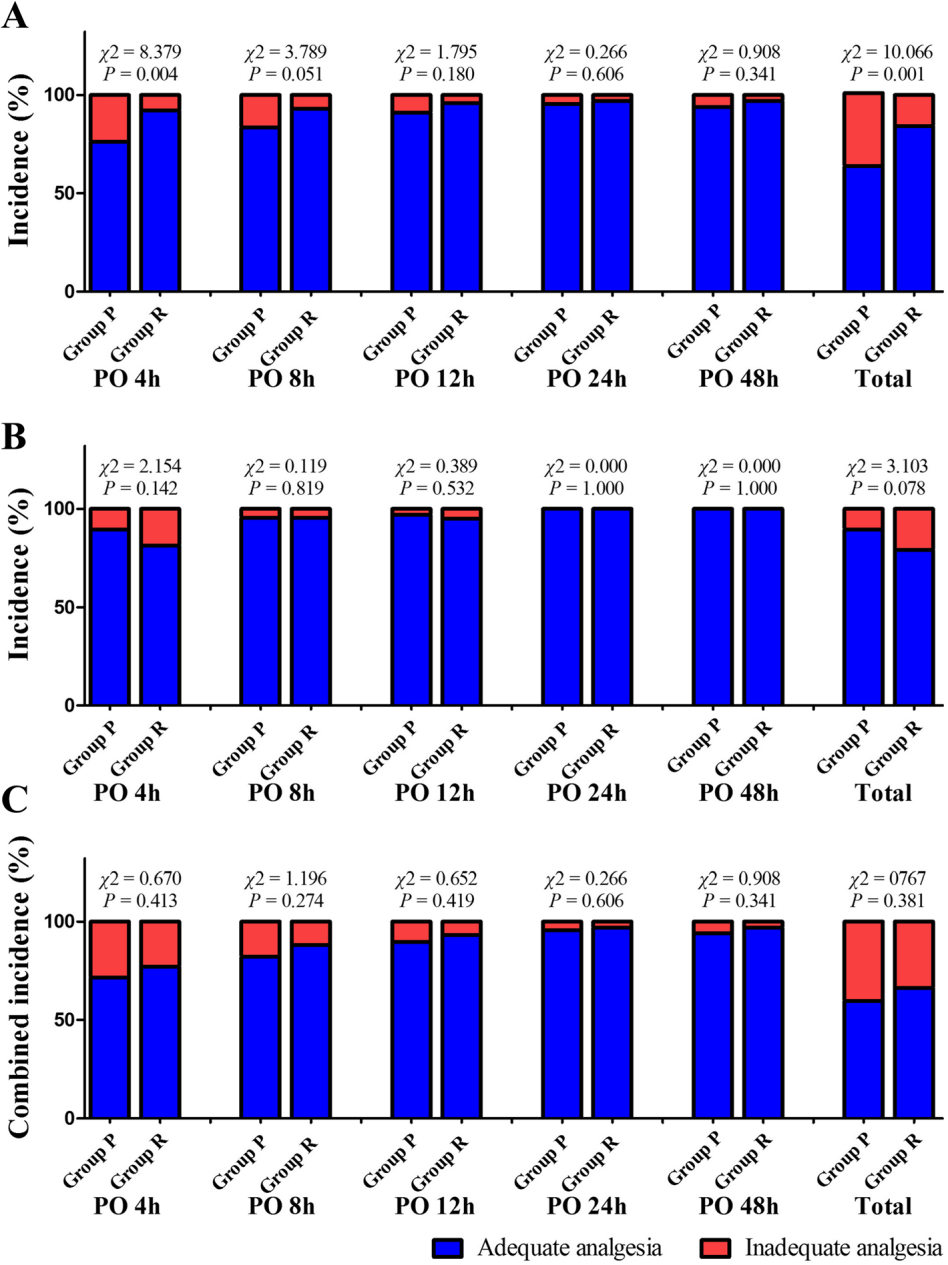
The incidence of postoperative inadequate treatment on incision pain (**a**), visceral pain (**b**) and the combined incidence (**c**) Groups P and R represent patients who underwent primary and repeated cesarean sections, respectively; VAS = visual analogue scale; PO = postoperative.

The distribution of pain VAS is shown in Fig. 2. The incidence of inadequate postoperative analgesia on incision or visceral pain at different times is shown in Fig. 3. In total, 24.4% (41/168) of patients were found to have inadequate treatment for their incision pain (Fig. 3a). The total incidence of inadequate analgesia on incision pain in group P was significantly higher than that in group R, and the RR for multiparas to experience inadequate analgesia on incision pain was 0.42 (95% CI: 0.25 to 0.74; *P* = 0.001) compared to primiparas. As shown in Fig. 3b, the total incidence of inadequate analgesia on visceral pain in group P was lower than that in group R, and the RR for patients in group R to experience inadequate analgesia on visceral pain was 1.75 (95% CI: 0.82 to 3.70; *P* = 0.078) compared to that for patients in group P. In addition, no significant difference was found in the total combined incidence of inadequate analgesia between groups P and R (Fig. 3c).

The results of subgroup analysis showed that group R was associated with a lower incidence of inadequate control on incision pain in both age groups (≤30 and > 30 years; RR, 0.47 [0.23 to 0.98], *P* = 0.033 and 0.40 [0.17 to 0.96], *P* = 0.042, respectively, Table 3). Group R was associated with a higher incidence of inadequate control on viscera pain in the age group ≤30 years (RR, 3.56 [1.05 to 12.04], *P* = 0.025).

**Table 3:** Subgroup analysis for different age groups

Age group	Outcomes	Group P	Group R	Statistics
≤30 years	Inadequate control on incision pain	18 (38.3%)	8 (18.2%)	χ2 = 4.506, *P =* 0.033
	Inadequate control on viscera pain	3 (6.4%)	10 (22.7%)	χ2 = 4.958, *P =* 0.025
	Inadequate control on both incision and viscera pain	18 (38.3%)	16 (36.4%)	χ2 = 0.036, *P =* 0. 849
> 30 years	Inadequate control on incision pain	7 (35.0%)	8 (14.0%)	χ2 = 4.149, *P =* 0.042
	Inadequate control on viscera pain	5 (25.0%)	11 (19.3%)	χ2 = 0.292, *P =* 0. 588
	Inadequate control on both incision and viscera pain	9 (45.0%)	18 (31.6%)	χ2 = 1.171, *P =* 0. 279

Group P and R mean patients who received primary and repeated cesarean delivery, respectively; Data were presented as numbers (percentage)

After propensity score matching according to preoperative factors, including age, gestational age, and preoperative complications, no significant differences remained between the two groups, and a total of 45 pairs of subjects were included for comparison of other postoperative outcomes (Table 4). As shown in Table 5, the pain VAS score at different time points were listed, and the distributions were similar to that in the non-matched cohort. Furthermore, the RR in multiparas for inadequate analgesia on incision pain was 0.35 (95% CI: 0.15 to 0.79; *P* = 0.007) compared to primiparas in this matched cohort. There was no significant difference in the incidence of inadequate analgesia on visceral pain between the two groups (*P* > 0.05). In addition, there was no significant difference in the incidence of adverse effects between the two groups. No respiratory depression, excessive sedation, or agitation was found in the present study. In addition, no significant difference was found in the time elapsed prior to the onset of pain, early waking time, sleep quality, and PCIA administration between the two groups. The results showed the mean hospital stay for primiparas was longer than that for multiparas.

**Table 4:** The postoperative short-term outcomes in different groups after propensity score matching

Outcomes	Group P (*n* = 45)	Group R (*n* = 45)	Statistics
Age (year)	30.3 ± 4.3	30.8 ± 3.4	*t* = 0.555, *P* = 0.580
Gestational age (week)	38.6 ± 1.0	38.5 ± 0.7	*t* = 0.569, *P* = 0.571
Preoperative complications	5 (11.1%)	3 (6.7%)	χ2 = 0.548, *P =* 0.458
Time to feel pain (hour)	3 (2, 6)	4 (2, 7)	*U* = 0.858, *P* = 0.391
Early walking time (hour)	28.9 ± 8.8	28.5 ± 9.6	*t* = 0.213, *P* = 0.832
Nausea or vomiting	3 (6.7%)	4 (8.9%)	χ2 = 0.155, *P =* 0.693
Pruritus	2 (4.5%)	3 (6.7%)	χ2 = 0.212, *P =* 0.645
Sleep quality PO 0d (poor)	16 (35.6%)	13 (28.9%)	χ2 = 0.457, *P =* 0.498
Sleep quality PO 1d (poor)	5 (11.1%)	3 (6.7%)	χ2 = 0.548, *P =* 0.458
PCIA consumption (mL)	111.1 ± 36.0	110.9 ± 37.3	*t* = 0.026, *P* = 0.979
Hospital stays (day)	3.5 ± 1.1	3.0 ± 0.8	*t* = 2.513, *P* = 0.014

Group P and R mean patients who received primary and repeated cesarean delivery, respectively; Data were presented as means ± SD, median (interquartile range) or as numbers (percentage); *PO* = Postoperative; *PCIA* = Patient controlled intravenous analgesia

**Table 5:** The patients’ postoperative pain in different groups after propensity score matching

Outcomes	Group P (*n* = 45)	Group R (*n* = 45)	Statistics
Rest incision pain VAS at PO 4 h	18 (11, 20)	11 (7, 16)	*U* = 2.508, *P* = 0.012
Moving incision pain VAS at PO 4 h	29 (21, 39)	23 (13, 30)	*U* = 2.705, *P* = 0.007
Visceral pain VAS at PO 4 h	22 (6, 32)	27 (17, 36)	*U* = 1.690, *P* = 0.091
Rest incision pain VAS at PO 8 h	13 (9, 19)	9 (4, 13)	*U* = 2.423, *P* = 0.015
Moving incision pain VAS at PO 8 h	25 (21, 34)	22 (12, 29)	*U* = 1.922, *P* = 0.055
Visceral pain VAS at PO 8 h	21 (8, 28)	20 (11, 30)	*U* = 0.342, *P* = 0.732
Rest incision pain VAS at PO 12 h	12 (6, 15)	9 (3, 12)	*U* = 1.973, *P* = 0.048
Moving incision pain VAS at PO 12 h	24 (21, 32)	15 (10, 26)	*U* = 3.198, *P* = 0.001
Visceral pain VAS at PO 12 h	13 (0, 25)	11 (0, 24)	*U* = 0.470, *P* = 0.638
Rest incision pain VAS at PO 24 h	6 (0, 13)	4 (0, 6)	*U* = 1.482, *P* = 0.138
Moving incision pain VAS at PO 24 h	22 (17, 29)	14 (9, 20)	*U* = 3.408, *P* = 0.001
Visceral pain VAS at PO 24 h	0 (0, 13)	5 (0, 15)	*U* = 0.373, *P* = 0.709
Rest incision pain VAS at PO 48 h	0 (0, 8)	0 (0, 4)	*U* = 1.043, *P* = 0.297
Moving incision pain VAS at PO 48 h	20 (12, 27)	14 (10, 20)	*U* = 1.804, *P* = 0.071
Visceral pain VAS at PO 48 h	0 (0, 8)	0 (0, 5)	*U* = 0.096, *P* = 0.924
Inadequate analgesia on incision pain	17 (37.8%)	6 (13.0%)	χ2 = 7.368, P = 0.007
Inadequate analgesia on visceral pain	7 (15.6%)	9 (19.6%)	χ2 = 0.252, *P* = 0.615

Group P and R mean patients who received primary and repeated cesarean delivery, respectively; Data were presented as median (interquartile range) or as numbers (percentage); *PO* = Postoperative; *VAS* = Visual analogue scale

### Changes of serum leukocyte count and neutrophil count

**Fig. 4:**
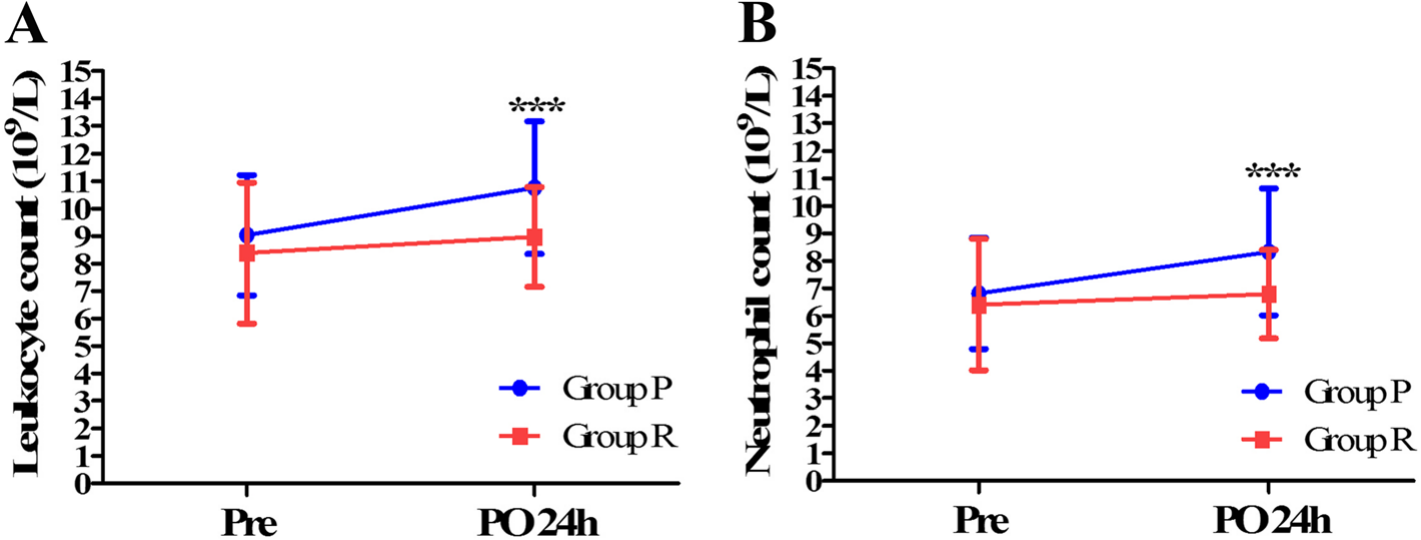
Changes in leukocyte count (**a**) and neutrophil count (**b**) before and after surgery. Means of groups P and R patients who underwent primary and repeated cesarean sections, respectively; Pre = preoperative; PO = postoperative; ^***^ compared to group R, *P* < 0.001.

Two-way repeated ANOVA for leukocyte count showed a group effect (*P* = 0.004), time effect (*P* < 0.001), and group and time interaction effect (*P* = 0.024). For the neutrophil count, the group effect (*P* = 0.012) and time effect (*P* < 0.001) were significant, while group and time interaction effects were not significant (*P* = 0.023). As shown in Fig. 4, there was no difference in the absolute leukocyte (9.03 ± 2.19 × 10^9^/L vs. 8.38 ± 2.57 × 10^9^/L, *P* = 0.202) and neutrophil (6.81 ± 2.02 × 10^9^/L vs. 6.41 ± 2.39 × 10^9^/L, *P* = 0.388) counts between the different groups before the surgery, while both leukocyte (10.76 ± 2.40 × 10^9^/L vs. 8.97 ± 1.81 × 10^9^/L, *P* < 0.001) and neutrophil (8.33 ± 2.31 × 10^9^/L vs. 6.78 ± 1.61 × 10^9^/L, *P* < 0.001) counts at 24 h after the surgery in group P were significantly higher than that in group R.

### Long-term follow-up

As shown in Table 6, no significant difference in pain status was found between the two groups 1 week after surgery. The results showed that at 4 weeks after surgery, the incidence of existing pain in group P was significantly higher than that in group R.

**Table 6:** The long-term postoperative outcomes in different groups after propensity score matching

Time point	Outcomes	Group P (*n* = 45)	Group R (*n* = 45)	Statistics
PO 1 week	Experiencing pain	38 (84.4%)	35 (77.7%)	χ2 = 0.653, *P =* 0.419
	location of pain (abdominal incision/viscera /both)	26 (57.8%)/5 (11.1%)/3 (6.7%)	26 (57.8%)/3 (6.7%)/0 (0.0%)	χ2 = 4.426, *P =* 0.219
	Affect sleep	14 (31.1%)	10 (22.2%)	χ2 = 0.909, *P =* 0.340
	Ability of daily life (partial dependency/fully autonomy)	19 (42.2%)/26 (57.8%)	18 (40.0%)/27 (60.0%)	χ2 = 0.046, *P =* 0.830
PO 4 week	Experiencing pain	28 (62.2%)	17 (37.7%)	χ2 = 6.403, *P =* 0.011
	location of pain (abdominal incision/ viscera/both)	18 (40.0%)/7 (15.6%)/1 (2.2%)	8 (17.8%)/4 (8.9%)/0 (0%)	χ2 = 9.434, *P =* 0.024
	Affect sleep	8 (17.8%)	4 (8.9%)	χ2 = 1.538, *P =* 0.215
	Ability of daily life (partial dependency/ fully autonomy)	45 (100%)/0 (0%)	45 (100%)/0 (0%)	χ2 = 0.000, *P =* 1.000

Group P and R mean patients who received primary and repeated cesarean delivery, respectively; Data were presented as numbers (percentage); *PO* = Postoperative

In additional, a significant difference was noted in the location of pain between patients in group P and group R.

## Discussion

Our results show that the total incidence of inadequate postoperative pain control was 36.3% using PCIA combined with hydromorphone and flurbiprofen, which was demonstrated as an effective combination for postoperative pain control [23]. One previous prospective cohort study [4] demonstrated that postoperative pain after a cesarean section reached 6 (interquartile range: 4 to 8), and the incidence of moderate to severe pain or requirement of extra analgesia was reported to range from 40 to 60% [5, 6]. Therefore, the analgesia strategy in this study might be effective for postoperative pain control. Nevertheless, the incidence of 36.3% remains relatively high, and more effective analgesia strategies should be explored in the future.

As we know, a high proportion of female patients are scheduled to undergo secondary cesarean section because of a previous cesarean section. In the United States, a repeat cesarean section due to a previous uterine scar contributed to more than 30% of all cesarean sections [24, 25]. Severe adhesions induced by previous surgery were often inevitable and thus, would cause higher operative difficulties [26, 27]. In this study, surgery duration in group R was significantly longer than that in group P, which is also indicative of higher operative difficulties in patients with repeat cesarean sections. In addition, previous surgery history might increase the patients’ pain sensitivity [28, 29]. Therefore, based on the above information, it was speculated that multiparas might experience more postoperative pain than primiparas.

For patients undergoing cesarean section, oxytocin, which can induce contraction pain, was routinely used to reduce intraoperative and postoperative hemorrhage [30, 31]. Thus, postoperative visceral pain induced by uterine contraction must frustrate the patients and should not be ignored. Although numerous previous studies have focused on the improvement of postoperative analgesia for cesarean section [32–35], many of these studies did not differentiate incision pain from visceral pain. However, a previous study found that the analgesic effects of the same analgesics on incision and uterine cramping pain varied [36]. Therefore, postoperative abdominal incision and visceral pain were evaluated in this study.

One previous study demonstrated that compared to primiparous women, the analgesic effect on post-cesarean uterine cramping pain is less in multiparous women [37]. The current results also showed that the incidence of inadequate treatment on visceral pain in group R was higher than that in group P, with the RR for multipara being 3.56 (95% CI: 1.05 to 12.04) in the patients aged ≤30 years. In addition, of all patients in the two groups, few were found to experience inadequate analgesia 8 h after the surgery, indicating that visceral pain might mainly appear at an early postoperative stage. Therefore, for the multipara, the focus should be on postoperative visceral pain at the early stage, especially for young patients.

In contrast, this study showed that multiparas were less likely to experience inadequate treatment on incision pain. The RR for multiparas was 0.42 (95% CI: 0.25 to 0.74), and the mean incision pain VAS score in group R was significantly lower than that in group P at several time points, including 4, 12, and 24 h after surgery. Based on the results of the current study, several reasons might account for this phenomenon. First, as shown in the study, the rate of preoperative complications in group P was higher than that in group R (80.6% vs. 53.5%) and was identified as a significant risk factor for inadequate treatment on incision pain. Second, through retrospective analysis, we found that both the leukocyte count and neutrophil count were significantly increased 24 h post-operation compared to that prior to surgery, and these elevations were higher in group P than in group R. Previous studies [38, 39] have also reported that there was a significant difference between preoperative and postoperative leukocyte and neutrophil counts for patients undergoing cesarean deliveries. However, it remains unclear whether this difference varied between primiparas and multiparas after cesarean deliveries. Increases in white blood cell and neutrophil counts have been demonstrated to be positively associated with inflammatory responses in previous studies [40–42]. Therefore, this indicated that different physiological responses to surgery or analgesia might exist between multiparas and primiparas. For primiparas, an effective analgesia strategy, e.g., combination of perioperative anti-inflammatory agents on incision pain should be considered.

In summary, because of the difference between postoperative control on visceral and incision pain, there was no significant difference in the combined incidence of inadequate analgesia on both types of pain between patients in groups P and R. Regarding the other postoperative outcomes during the hospital stay, no significant difference was found in the incidence of adverse events, time to feel pain, early walking time, sleep quality, and PCIA administration between the two groups. However, we found that the mean hospital stay for primiparas was longer than that for multiparas. This indicated that primiparas might need more care after cesarean section. Furthermore, the current study demonstrated that primiparas might experience a longer duration of pain, because higher incidences of existing pain and affected sleep were found in group P than in group R 4 weeks after surgery. This might be due to the higher incidence of inadequate incision pain control in patients of group P, because a previous study has identified inadequately controlled acute postoperative pain as a risk factor for the development of chronic pain post-operation [43].

Several limitations should be noted in the study. First, the study only included Chinese women from urban areas; thus, race and socio-economic status should be considered when interpreting the current results [44, 45]. Second, although a significant difference in postoperative pain status was found between primiparas and multiparas, the current sample size was relatively small. Third, in the current study, all multiparas were undergoing secondary surgery; thus, the differences for those who underwent two or more cesarean deliveries were not known. Thus, to address these potential limitations, a multicenter study with a larger sample size might be needed, and more studies including other populations should be performed in the future.

## Conclusion

Multiparas under 30 years of age may be more prone to experiencing moderate to severe visceral pain under PCIA with opioids during the first 48 h after surgery compared to primiparas; however, primiparas have a higher incidence of inadequate treatment on incision pain and possibly a higher incidence of existing pain 4 weeks after surgery. Based on the results of the current study, individual differences between primipara and multipara should be considered in postoperative analgesia in the future.

## Data Availability

All data can be acquired from the corresponding author (HL) by request.

## Abbreviations

ANOVA: analysis of variance
CI: confidence intervals
HADS: hospital anxiety and depression scale
OR: Odds ratios
PCIA: patient-controlled intravenous analgesia
RR: Relative Risk
SD: standard deviation
VAS: visual analogue scale
